# ﻿First records of the crane fly genus *Helius* Lepeletier & Serville (Diptera, Limoniidae) in Guangxi, China with description of one new species

**DOI:** 10.3897/zookeys.1168.104106

**Published:** 2023-06-27

**Authors:** Yuanyuan Xu, Shenglin Zhang, Guoquan Wang, Ding Yang, Xiao Zhang

**Affiliations:** 1 Guangxi key laboratory of Agric-Environment and Agric-Products Safety and National Demonstration Center for Experimental Plant Science Education, Agricultural College, Guangxi University, Nanning 530004, China Guangxi University Nanning China; 2 Shandong Engineering Research Center for Environment-Friendly Agricultural Pest Management, College of Plant Health and Medicine, Qingdao Agricultural University, Qingdao 266109, China Qingdao Agricultural University Qingdao China; 3 College of Plant Protection, China Agricultural University, Beijing 100193, China China Agricultural University Beijing China

**Keywords:** Chinese fauna, distribution, Elephantomyiini, new record, taxonomy

## Abstract

The genus *Helius* Lepeletier & Serville, 1828 is recorded in Guangxi, China for the first time with the following three species belonging to the subgenus H. (Helius): H. (H.) damingshanus**sp. nov.**, H. (H.) nipponensis (Alexander, 1913) and H. (H.) stenorhynchus stenorhynchus Alexander, 1954. Among them, H. (H.) stenorhynchus stenorhynchus is also a new record in China. Their descriptions and illustrations, as well as the first key to Chinese H. (Helius) crane flies, are presented.

## ﻿Introduction

*Helius* Lepeletier & Serville, 1828 (in Latreille et al. 1828) is a genus of the family Limoniidae with 231 species and subspecies (Oosterbroek 2023). *Helius* crane flies are separated into nine subgenera, of which H. (Helius) is the species-richest subgenus with 180 known species and subspecies distributed throughout the Oriental (71 species and subspecies), Neotropical (50 species and subspecies), Afrotropical (23 species), Palaearctic (21 species and subspecies), Australasian (15 species) and Nearctic (2 species) regions (Oosterbroek 2023).

A total of 21 H. (Helius) species have been recorded in China, of which 18 were described by C. P. Alexander between 1913–1945 (Alexander 1913, 1924, 1928, 1929a, 1929b, 1930a, 1930b, 1932b, 1932c, 1932d, 1937, 1938, 1940a, 1940b, 1945), two were described by F. W. Edwards in 1916 and 1921 respectively, and one was described by E. Brunetti in 1912. These species are known to be distributed in Taiwan (11 species), Sichuan (4 species), Jiangxi (2 species), Zhejiang (2 species), Fujian (1 species), Guangdong (1 species) and Jilin (1 species) (Oosterbroek 2023). No new species from China have been described for more than 70 years, and more taxonomic and geographical studies of *Helius* crane flies in China need to be carried out.

Guangxi Zhuang Autonomous Region (Guangxi) is located at the southeast edge of the Yunnan-Guizhou Plateau in China, with mountains, hills, platforms, plains and other types of landforms. There are many hills and plains in the middle and south of Guangxi, a basin-like region called “Guangxi Basin”. Although Guangxi is a region with high biodiversity, there is no record of *Helius* crane flies. To improve the understanding of the diversity of crane flies in Guangxi and the distribution of *Helius* in China, some investigations on crane flies in Guangxi have been initiated by the authors together with other entomologists since 2011, resulting in the discovery of *Helius* in Guangxi for the first time.

## ﻿Material and methods

**Figure 1.**
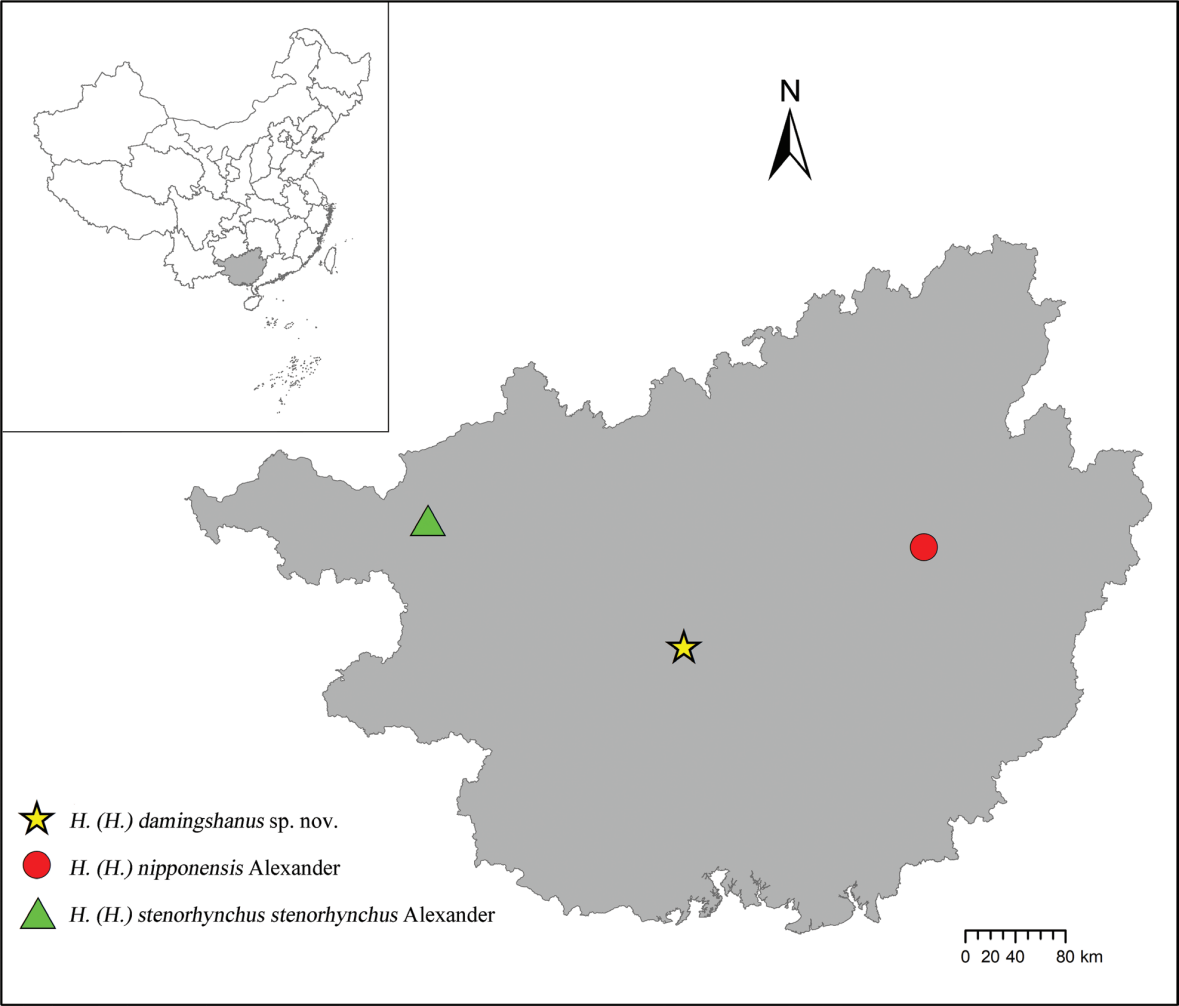
Collecting sites of H. (Helius) crane flies in Guangxi, China in this study.

Specimens for this study were collected from several localities in Guangxi, China (Fig. 1) by different entomologists during 2013–2015 and are deposited in the Entomological Museum of China Agricultural University, Beijing, China (CAU). Genitalic preparation of males was made by macerating the apical portion of the abdomen in cold 10% sodium hydroxide (NaOH) for 12–15 hours. Observations and illustrations were made using a ZEISS Stemi 2000–C stereomicroscope. Photographs were taken with a Canon EOS 90D digital camera through a Canon EF100 mm f/2.8L Macro IS USM lens.

The morphological terminology mainly follows Cumming and Wood (2017), while that for wing venation follows de Jong (2017). The following abbreviations in figures are used: **A_1_** = first branch of anal vein, **aed** = aedeagus, **C** = costal vein, **cerc** = cercus, **CuA** = anterior branch of cubital vein, **CuP** = posterior branch of cubital vein, **dm** = discal medial cell, **ea** = ejaculatory apodeme, **goncx** = gonocoxite, **hyp vlv** = hypogynial valve, **i gonst** = inner gonostylus, **interb** = interbase, **M** = medial vein, or media, **M_1_** = first branch of media, **M_3_** = third branch of media, **M_4_** = fourth branch of media, **m-cu** = media-cubital crossvein, **m-m** = medial crossvein, **o gonst** = outer gonostylus, **pm** = paramere, **R** = radius, or radial vein, **R_1_** = anterior branch of radius, **R_4_** = upper branch of third branch of radius, **R_5_** = lower branch of third branch of radius, **r-m** = radial-medial crossvein, **Rs** = radial sector, **Sc** = subcostal vein, **sc-r** = subcostal-radial crossvein, **st** = sternite, **tg** = tergite.

## ﻿Taxonomy

### ﻿Key to Chinese species of Helius (Helius)

**Table:** 

1	Rostrum about equal in length to combined head (excluding rostrum) and thorax (Fig. 7b)	**2**
–	Rostrum about equal in length to remainder of head (Figs 2b, 5b)	**5**
2	Wing with cell dm open by absence of m-m (Alexander 1929b)	**Helius (Helius) liliputanus Alexander, 1929**
–	Wing with cell dm closed	**3**
3	Wing with Sc atrophied (Fig. 7d)	**Helius (Helius) stenorhynchus stenorhynchus Alexander, 1954**
–	Wing with Sc not atrophied (Figs 2d, 5d)	**4**
4	Prescutum and presutural scutum brownish black with humeral region vaguely brightened. Femora of legs black with bases yellowish (Alexander 1932b)	**Helius (Helius) pluto (Alexander, 1932)**
–	Prescutum and presutural scutum shiny ferruginous without markings. Femora of legs yellowish with tips narrowly blackened (Fig. 7a) (Alexander 1928)	**Helius (Helius) rufithorax Alexander, 1928**
5	Wing nearly hyaline to yellow, with stigma indistinct or missing (Fig. 5d)	**6**
–	Wing pale brown to dark brown, with stigma obvious, brown or dark brown (Figs 2d, 7d)	**11**
6	Prescutum and presutural scutum with markings	**7**
–	Prescutum and presutural scutum without markings	**8**
7	Prescutum and presutural scutum with a narrow median line (Brunetti 1912). Outer gonostylus with subtip not expanded. Interbase in the shape of pale scoop with stem short and stout (Alexander 1964)	**Helius (Helius) unicolor (Brunetti, 1912)**
–	Prescutum and presutural scutum with three broad stripes (Fig. 5c). Outer gonostylus with subtip slightly expanded (Fig. 6a, b, e). Interbase horn-like (Fig. 6c, d, f)	**Helius (Helius) nipponensis (Alexander, 1913)**
8	Head generally pale gray or gray. Prescutum and presutural scutum pale yellow	**9**
–	Head generally blackish brown. Prescutum and presutural scutum dark brown	**10**
9	Antenna uniformly brown. Leg with femora yellow, tips narrowly darkened (Fig. 7a), tibiae and tarsi pale yellow, outer tarsal segments brown. Wing with distance between tips of R_4_ and R_5_ about 5 times as long as distance between tips of R_1_ and R_4_, Sc ending at middle of Rs (Alexander 1932c)	**Helius (Helius) anaemicus Alexander, 1932**
–	Antenna brownish black with scape yellowish. Leg with femora yellow, remaining segments passing into brownish yellow. Wing with distance between tips of R_4_ and R_5_ about 3 times as long as distance between tips of R_1_ and R_4_, Sc ending shortly before fork of Rs (Alexander 1930b)	**Helius (Helius) pallidissimus Alexander, 1930**
10	Antenna as long as head (including rostrum). Posterior margin of tergite 9 with two conspicuous hairy points. Gonostyli broad, outer gonostylus with a double membranous lobe (Edwards 1921)	**Helius (Helius) barbatus Edwards, 1921**
–	Antenna not much longer than rostrum. Posterior margin of tergite 9 without hairy points. Gonostyli slender, outer gonostylus without lobe (Edwards 1916)	**Helius (Helius) nigriceps (Edwards, 1916)**
11	Wing with conspicuous brown seams along cord and CuA (Alexander 1924)	**Helius (Helius) subfasciatus Alexander, 1924**
–	Wing without such seams	**12**
12	Prescutum and presutural scutum with markings	**13**
–	Prescutum and presutural scutum without markings	**17**
13	Wing with tip broadly darkened (Alexander 1938)	**Helius (Helius) polionotus Alexander, 1938**
–	Wing with tip not broadly darkened	**14**
14	Wing with distance between tips of R_4_ and R_5_ shorter than distance between tips of R_1_ and R_4_ (Alexander 1937)	**Helius (Helius) aciferus Alexander, 1937**
–	Wing with distance between tips of R_4_ and R_5_ longer than distance between tips of R_1_ and R_4_ (Figs 2d, 5d, 7d)	**15**
15	Gonocoxite of hypopygium with a spinous lobe (Alexander 1929a)	**Helius (Helius) tenuistylus Alexander, 1929**
–	Gonocoxite of hypopygium without spinous lobe	16
16	Wing with distance between tips of R_4_ and R_5_ about 1.5 times as long as distance between tips of R_1_ and R_4_. Abdominal sternites bicolored (Alexander 1937). Outer gonostylus with inner spine acute apically (Fig. 4j)	**Helius (Helius) haemorrhoidalis Alexander, 1937**
–	Wing with distance between tips of R_4_ and R_5_ about 3 times as long as distance between tips of R_1_ and R_4_ (Fig. 2d). Abdominal sternites not bicolored. Outer gonostylus with inner spine flat apically (Fig. 3e)	**Helius (Helius) damingshanus sp. nov.**
17	Wing with m-cu from shortly before to just beyond fork of M	**18**
–	Wing with m-cu more than 1/4 its length beyond fork of M (Figs 2d, 5d, 7d)	**20**
18	Gonocoxite of hypopygium with a conspicuous spiniferous lobe, tip of outer gonostylus with a very indistinctly tooth (Alexander 1932d)	**Helius (Helius) infirmu s Alexander, 1932**
–	Gonocoxite of hypopygium without lobe, tip of outer gonostylus bispinous	**19**
19	Legs with femora and tibiae brownish black, tarsi pale to brownish yellow. Outgrowth of interbase round apically (Fig. 4d), aedeagus with distal half spiral (Alexander 1929a)	**Helius (Helius) attenuatus Alexander, 1929**
–	Legs with femora and tibiae bright yellow, terminal tarsal segments darkened. Outgrowth of interbase acute apically (Fig. 4g), aedeagus with tip bent (Alexander 1940a)	**Helius (Helius) franckianus Alexander, 1940**
20	Interbase of hypopygium with a small pale lobe at outer margin near base (Fig. 4q), aedeagus spiral (Alexander 1940b)	**Helius (Helius) tienmuanus Alexander, 1940**
–	Interbase of hypopygium without lobe at outer margin near base, aedeagus straight	**21**
21	Wing strongly tinged with brown, stigma and wing apex in outer radial field darker, prearcular and costal fields yellowish brown (Alexander 1945)	**Helius (Helius) lienpingensis Alexander, 1945**
–	Wing with a brownish tinge, stigma darker	**22**
22	Interbase of hypopygium with apical point bent across blade (Alexander 1930a)	**Helius (Helius) minusculus Alexander, 1930**
–	Interbase of hypopygium with apical point not bent across blade (Alexander 1930a)	**Helius (Helius) chikurinensis Alexander, 1930**

### ﻿Class Insecta Linnaeus, 1758

**Order Diptera Linnaeus, 1758**

**Family Limoniidae Speiser, 1909**

**Subfamily Limoniinae Speiser, 1909**

#### ﻿Genus Helius

Taxon classificationAnimaliaDipteraLimoniidae

Lepeletier & Serville, 1828

C94FED36-A242-545A-B991-972C318F6791

Megarhina Lepeletier & Serville, 1828 (in Latreille et al. 1828: 585). Type-species: Limnobialongirostris Meigen, 1818 (monotypic)
Leptorhina Stephens, 1829: 243. Type-species: Limnobialongirostris Meigen, 1818 (monotypic).
Rhamphidia Meigen, 1830: 281. Type-species: Limnobialongirostris Meigen, 1818 (designated in Macquart 1834).

##### Note.

As an unjustified new name for *Megarhina*, *Helius* was adopted for stability (Sabrosky 1999; Oosterbroek 2023).

#### ﻿ Helius (Helius) damingshanus

Taxon classificationAnimaliaDipteraLimoniidae

Xu, Zhang & Zhang sp. nov.

D8320EB4-40A0-56FB-B16D-39C256AC177E

https://zoobank.org/5B3DB193-FF08-4B9E-B0B0-EEC31B1F5ADB

Figs 2 , 3

##### Type material.

***Holotype***: China • ♂; Guangxi Zhuang Autonomous Region, Wuming County, Mount Damingshan; 11 May 2014; Xiumei Lu leg.; CAU. ***Paratypes***: China • 2 ♂, 1 ♀; same data as holotype; CAU.

##### Diagnosis.

Antenna with basal flagellomeres oval. Rostrum about equal in length to remainder of head. Prescutum and presutural scutum pale brown with a narrow darker median line. Femora of legs pale brown with base paler. Wing with oval brown stigma; Sc ending shortly before fork of Rs; m-cu beyond fork of M. Outer gonostylus slightly curved, inner spine flat apically. Interbase nearly globular, laterally with narrow, apically dilated outgrowth. Distal 2/3 of aedeagus arched dorsally.

##### Description.

**Figure 2.**
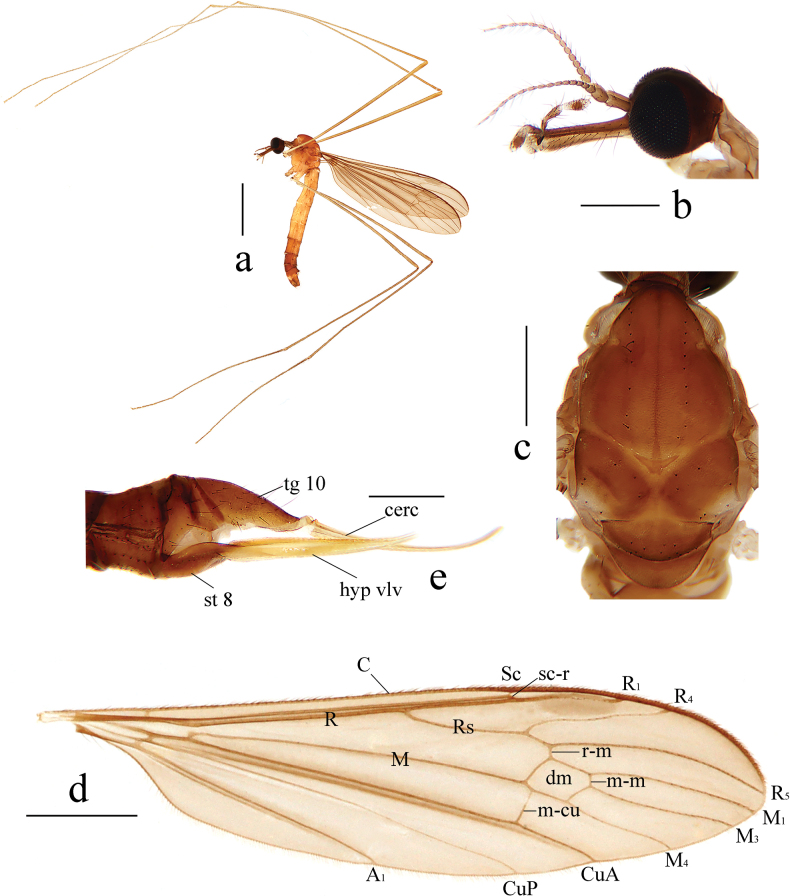
Helius (Helius) damingshanus sp. nov. **a** habitus of male, lateral view **b** male head, lateral view **c** male thorax, dorsal view **d** male wing **e** female ovipositor, lateral view. Scale bars: 2.0 mm (**a**); 0.5 mm (**b, c**); 1.0 mm (**d**); 0.4 mm (**e**).

**Male** (Fig. 2a). Body length 5.6–6.0 mm (excluding rostrum), wing length 6.5–6.9 mm, rostrum length 0.5–0.6 mm, antenna length 1.1–1.2 mm.

***Head*** (Fig. 2b). Dark brown. Setae on head brownish black. Antenna brown. Scape long cylindrical, 2.5 times as long as wide; pedicel oval; basal flagellomeres oval, with short brownish black verticils, outer flagellomeres tapering apically and elongated, with long brownish black verticils that exceed length of corresponding flagellomere. Rostrum about equal in length to remainder of head, brown with brownish black setae. Palpus brown with brownish black setae.

***Thorax*** (Fig. 2c). Pronotum brown. Prescutum and presutural scutum pale brown with a narrow darker median line. Postsutural scutum pale brown, each lobe with a white spot. Scutellum brown, paler medially. Mediotergite pale brown. Pleuron (Fig. 2a) brownish yellow with anepisternum darker. Setae on thorax brownish black. Fore and mid coxae brownish yellow, hind coxa pale yellow; trochanters pale brownish yellow, tips narrowly black; femora pale brown with base paler; tibiae and tarsi pale brown. Setae on legs brownish black. Wing (Fig. 2d) tinged with pale brown. Stigma oval and brown. Veins brown. Venation: Sc long, ending slightly before fork of Rs; sc-r close to tip of Sc; distance between tips of R_4_ and R_5_ about 3 times as long as distance between tips of R_1_ and R_4_; cell dm short, about 1.5 times as long as wide; m-cu variable in position, from fork of M to 1/4 of cell dm. Halter brown with knob brownish yellow.

***Abdomen*** (Fig. 2a). First abdominal segment short, brownish yellow, segments 2–5 yellow, segment 6 brownish yellow, segments 7–9 brown. Setae on abdomen brownish black.

**Figure 3.**
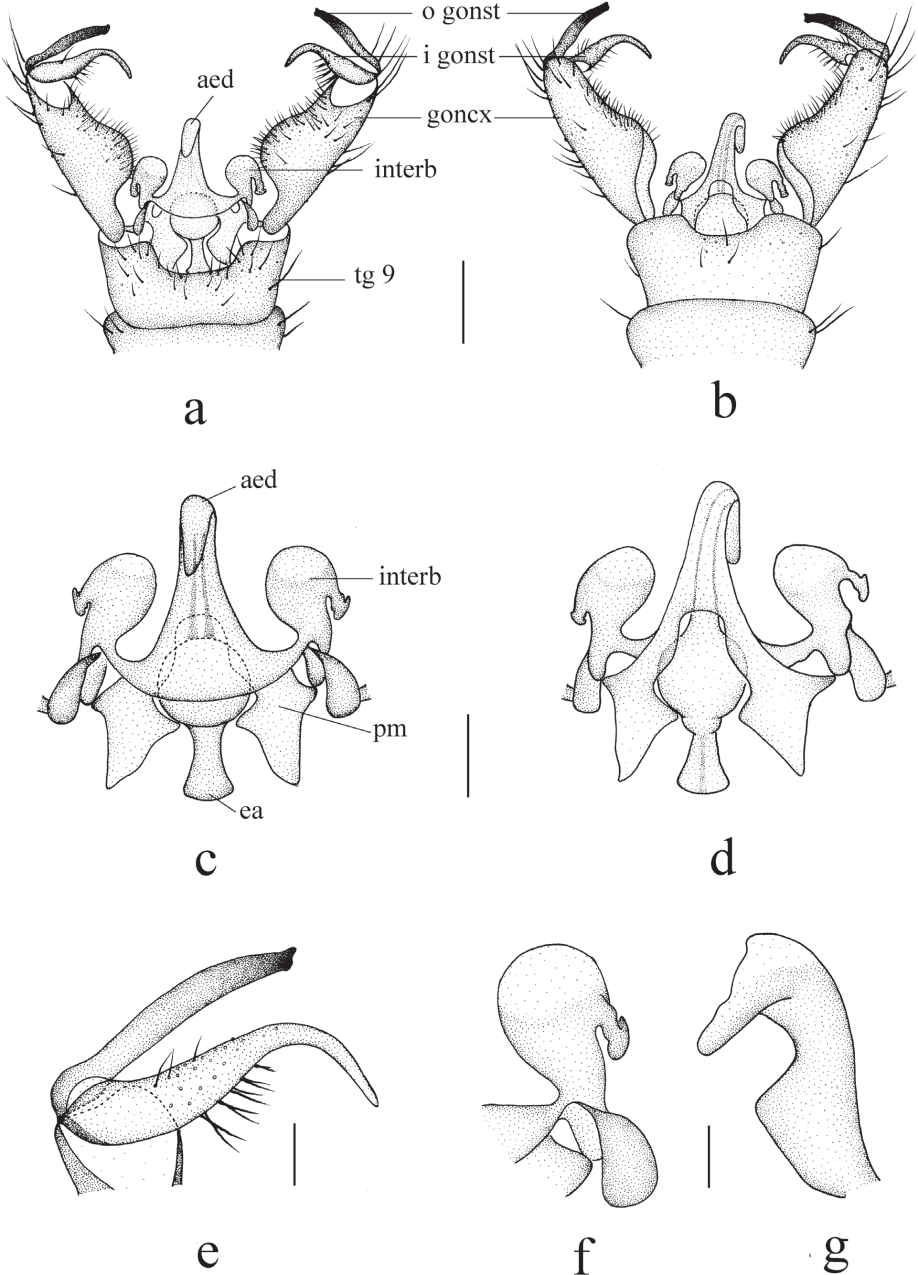
Helius (Helius) damingshanus sp. nov. **a** male hypopygium, dorsal view **b** male hypopygium, ventral view **c** aedeagal complex, dorsal view **d** aedeagal complex, ventral view **e** gonostyli, dorsal view **f** interbase, dorsal view **g** interbase, lateral view. Scale bars: 0.2 mm (**a, b**); 0.1 mm (**c, d**); 0.05 mm (**e, f, g**).

***Hypopygium*** (Fig. 3). Brown. Posterior margin of tergite 9 with a broad U-shaped emargination (Fig. 3a). Gonocoxite nearly cylindrical, base narrowed, inner margin distinctly swollen, produced into rounded protrusion, outer side with long brown setae (Fig. 3a, b). Outer gonostylus (= clasper of gonostylus in Ribeiro 2006) slightly curved, distal half blackened; tip weakly bispinous, inner spine flat apically (Fig. 3a, b, e). Inner gonostylus (= lobe of gonostylus in Ribeiro 2006) arched, broad at base, gradually narrowing towards apex, middle of inner side with long brown setae (Fig. 3a, b, e). Aedeagal complex with semen pump spherical (Fig. 3c, d); ejaculatory apodeme elongated, widened at base (Fig. 3c, d); aedeagus wide at base, distal 2/3 arched dorsally (Fig. 3a–d). Parameres apically fused, basal parts flattened, in the shape nearly triangular plate (Fig. 3c, d). Interbase globular laterally, producing into narrow, apically dilated outgrowth, beak-shaped in lateral aspect (Fig. 3c, d, f, g).

**Female.** Body length 8.0 mm (excluding rostrum), wing length 6.5 mm, rostrum length 0.5 mm. Generally similar to male by body coloration. Ovipositor (Fig. 2e) with tergite 10 brown. Cercus pale brownish yellow, long and slender, distal part curved dorsally, tip acute. Sternite 8 brown. Hypogynial valve pale brownish yellow, long and slender, tip reaching approximately middle of cercus.

##### Etymology.

The specific name refers to the type locality Mount Damingshan.

##### Distribution.

China (Guangxi).

##### Remarks.

**Figure 4.**
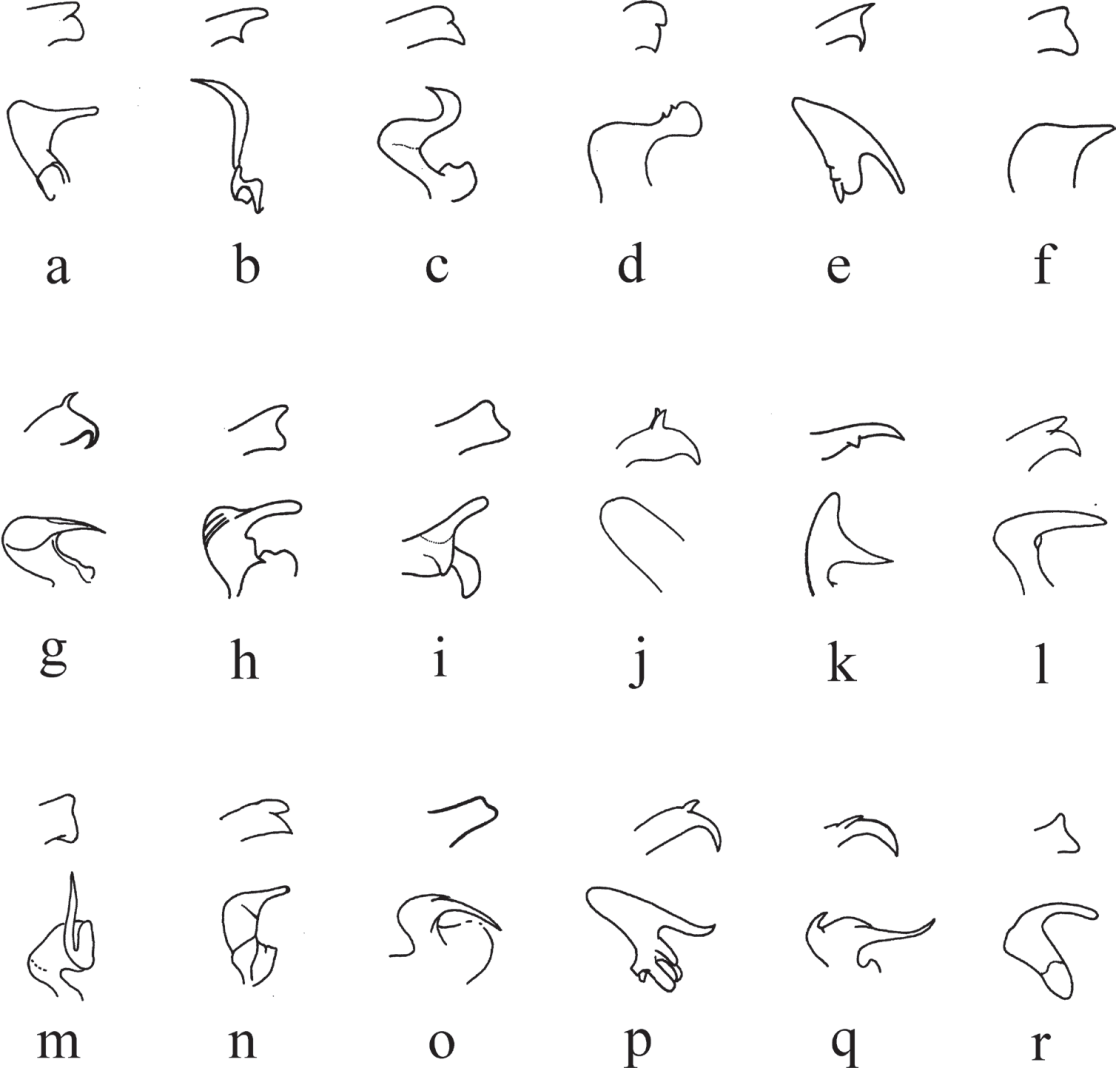
Tips of outer gonostyli (upper) and interbases (lower) of some Palaearctic and Oriental Helius (Helius) species **a**H. (H.) anamalaiensis Alexander, 1967 **b**H. (H.) apophysalis Alexander, 1967 **c**H. (H.) arunachalus Alexander, 1975 **d**H. (H.) attenuatus (Alexander, 1929) **e**H. (H.) catreus Alexander, 1967 **f**H. (H.) costosetosus Alexander, 1932 **g**H. (H.) franckianus (Alexander, 1940) **h**H. (H.) fuscoangustus Alexander, 1967 **i**H. (H.) garcianus Alexander, 1972 **j**H. (H.) haemorrhoidalis (Alexander, 1937) **k**H. (H.) oxystylus (Alexander, 1967) **l**H. (H.) perflavens Alexander, 1964 **m**H. (H.) pluto (Alexander, 1932) **n**H. (H.) serenus Alexander, 1967 **o**H. (H.) tanyrhinus Alexander, 1964 **p**H. (H.) taos Alexander, 1967 **q**H. (H.) tienmuanus (Alexander, 1940) **r**H. (H.) verticillatus Alexander, 1967. After Alexander 1929a (**d**), 1932a (**f**), 1932b (**m**), 1937 (**j**), 1940a (**g**), 1940b (**q**), 1964 (**l, o**), 1967 (**a, b, e, h, k, n, p, r**), 1972 (**i**), 1975 (**c**).

Similar to many Palaearctic and Oriental species, H. (H.) damingshanus sp. nov. has also a simple gonocoxite and curved aedeagus, but can be distinguished from its congeners by the details of the outer gonostylus and the interbase (Fig. 4). In H. (H.) apophysalis Alexander, 1967, H. (H.) haemorrhoidalis, H. (H.) oxystylus (Alexander, 1967), H. (H.) taos Alexander, 1967 and H. (H.) tienmuanus, the size of the two spines at the tip of the outer gonostylus is significantly unequal (Fig. 4b, j, k, p, q), while in H. (H.) catreus Alexander, 1967 and H. (H.) franckianus, the spines are almost equal in size, noticeably thin and pointed (Fig. 4e, g). In the remaining species, including the new species, the two spines are relatively stout and the difference in their sizes is not very noticeable, but these species can be distinguished by the tip of the outgrowth of the interbase, which is elongated and with acute apex in H. (H.) arunachalus Alexander, 1975, H. (H.) pluto and H. (H.) tanyrhinus Alexander, 1964 (Fig. 4c, m, o), short and obtuse in H. (H.) costosetosus Alexander, 1932 and H. (H.) perflavens Alexander, 1964 (Fig. 4f, l), and rounded in H. (H.) anamalaiensis Alexander, 1967, H. (H.) attenuatus, H. (H.) fuscoangustus Alexander, 1967, H. (H.) garcianus Alexander, 1972, H. (H.) serenus Alexander, 1967 and H. (H.) verticillatus Alexander, 1967 (Fig. 4a, d, h, i, n, r).

Only H. (H.) attenuatus and the new species have similar outgrowth of interbase with tip flattened (Figs 3f, g, 4d), but the new species have tip nearly beak-shaped in lateral aspect while H. (H.) attenuatus has tip rounded. Helius (H.) damingshanus sp. nov. can be also distinguished by the pale brown prescutum and presutural scutum having a narrow median darker line (Fig. 2c), the brownish yellow abdomen with darker terminal segments (Fig. 2a), and the inner spine of the outer gonostylus being flat apically (Fig. 3e). In H. (H.) attenuatus, the prescutum and presutural scutum is uniformly brownish black, the abdomen is uniformly black (Alexander 1929a), and the inner spine of the outer gonostylus is acute apically (Fig. 4d). In addition, in H. (H.) attenuatus, the body color is generally darker (Alexander 1929a), and the outgrowth of the interbase is more expanded (Fig. 4d).

#### ﻿ Helius (Helius) nipponensis

Taxon classificationAnimaliaDipteraLimoniidae

(Alexander, 1913)

206AA9C3-953F-567B-B062-B5618012A1A3

Figs 5 , 6

Rhamphidia nipponensis Alexander, 1913: 207. Type locality: Japan, Tokyo.
Rhamphidia nipponensis : Alexander 1920: 8.
Helius nipponensis : Alexander 1929b: 532.
Helius (Helius) nipponensis : Podenas et al. 2015: 78.

##### Specimens examined.

China • 1 ♂, 1 ♀; Guangxi Zhuang Autonomous Region, Jinxiuyao Autonomous County, Mount Dayaoshan, Silver Fir Park; 1170 m a.s.l.; 21 July 2015; Yan Li leg.; light trap; CAU.

##### Diagnosis.

Antenna with basal flagellomeres cylindrical. Rostrum about equal in length to remainder of head. Prescutum and presutural scutum brownish yellow with three broad brown stripes; median stripe longest, broadest, darker in front; lateral stripes extending onto lobes of postsutural scutum. Femora of legs brownish yellow to brown. Wing with stigma very indistinct; Sc ending near fork of Rs; m-cu beyond fork of M. Outer gonostylus curved with subtip slightly expanded; outer spine small, inner spine large and bent outwards. Interbase horn-like. Aedeagus straight.

##### Description.

**Figure 5.**
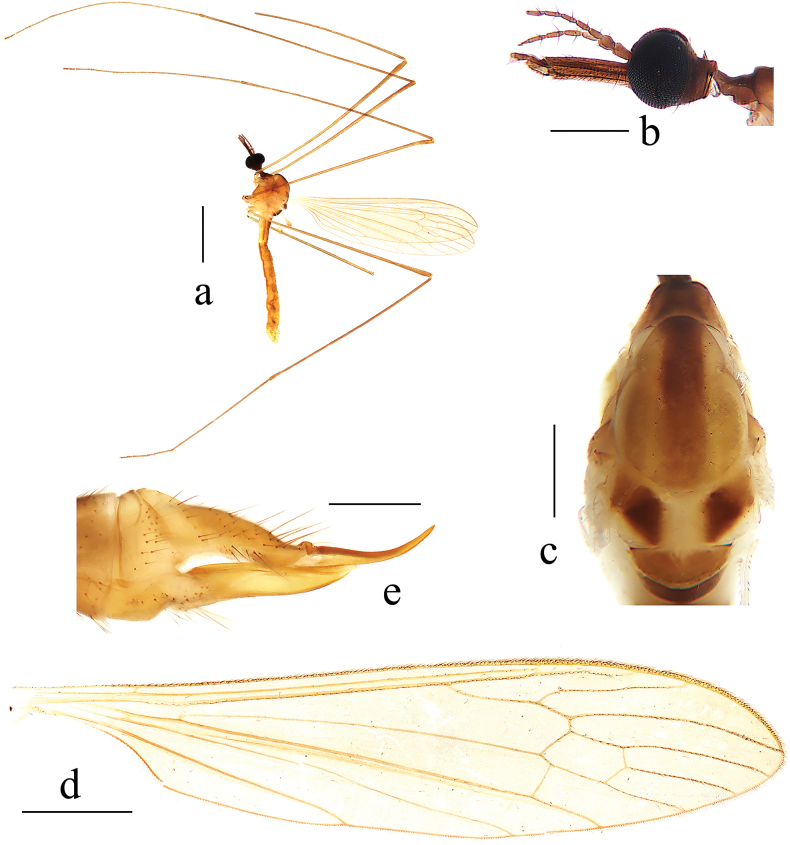
Helius (Helius) nipponensis (Alexander, 1913) **a** habitus of male, lateral view **b** male head, lateral view **c** male thorax, dorsal view **d** male wing **e** female ovipositor, lateral view. Scale bars: 2.0 mm (**a**); 0.5 mm (**b, c**); 1.0 mm (**d**); 0.4 mm (**e**).

**Male** (Fig. 5a). Body length 6.7 mm (excluding rostrum), wing length 7.0 mm, rostrum length 0.5 mm.

***Head*** (Fig. 5b). Dark brown. Setae on head brownish black. Antenna brown. Scape long cylindrical, twice as long as wide; pedicel oval; flagellomeres cylindrical, verticils brownish black, not exceeding flagellomere in length. Rostrum about equal in length to remainder of head, brown with brownish black setae. Palpus pale brown with dark brown setae.

***Thorax*** (Fig. 5c). Pronotum dark brown with two sides brownish yellow. Prescutum and presutural scutum brownish yellow with three broad brown stripes; median stripe longest, broadest, darker in front; lateral stripes extending onto lobes of postsutural scutum. Postsutural scutum dark brown, middle area pale brownish yellow, each lobe with a yellow spot. Scutellum brown with margins paler. Mediotergite dark brown. Pleuron (Fig. 5a) brownish yellow with anepisternum brown. Setae on thorax dark brown. Fore and mid coxae pale brownish yellow, hind coxa yellow; trochanters pale brownish yellow, tips narrowly black; femora brownish yellow to brown; tibiae and tarsi brown. Setae on legs dark brown. Wing (Fig. 5d) tinged with yellow. Stigma very indistinct. Veins pale brown. Venation: Sc long, ending opposite fork of Rs; sc-r close to tip of Sc; distance between tips of R_4_ and R_5_ about 2.5 times as long as distance between tips of R_1_ and R_4_; cell dm about twice as long as wide; m-cu more than 1/3 its length beyond fork of M, near 1/4 of cell dm. Halter pale yellow with knob darker.

***Abdomen*** (Fig. 5a). Tergites dark brownish yellow. Sternites brownish yellow with sternite 1 paler. Setae on abdomen brown.

**Figure 6.**
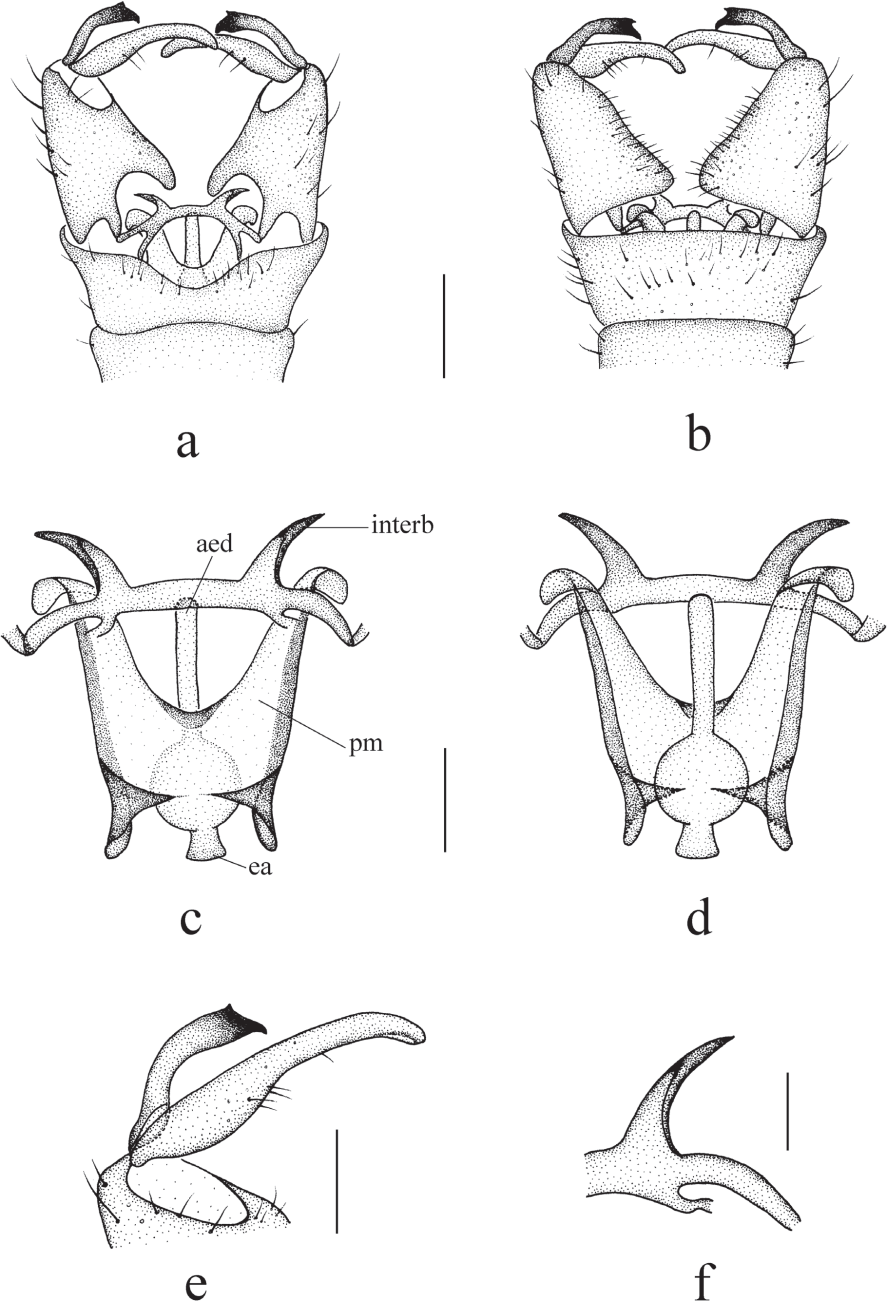
Helius (Helius) nipponensis (Alexander, 1913) **a** male hypopygium, dorsal view **b** male hypopygium, ventral view **c** aedeagal complex, dorsal view **d** aedeagal complex, ventral view **e** gonostyli, dorsal view **f** interbase, dorsal view. Scale bars: 0.2 mm (**a, b**); 0.1 mm (**c, d, e**); 0.05 mm (**f**).

***Hypopygium*** (Fig. 6). Yellow. Posterior margin of tergite 9 with a V-shaped emargination (Fig. 6a). Gonocoxite conical, outer side with long brown setae (Fig. 6a, b). Outer gonostylus curved with subtip slightly expanded; tip blackened and bispinous, outer spine small, inner spine large and bent outwards (Fig. 6a, b, e). Inner gonostylus curved, broad at basal half (Fig. 6a, b, e). Aedeagal complex with semen pump spherical (Fig. 6c, d), ejaculatory apodeme short and flattened (Fig. 6c, d); aedeagus straight, rod-shaped (Fig. 6a–d). Parameres medially fused and expanded, basal parts short, apical elongated with tip bent outwards. Interbase horn-like (Fig. 6c, d, f).

**Female.** Body length 7.3 mm (excluding rostrum), wing length 6.5 mm, rostrum length 0.4 mm. Generally similar to male by body coloration. Ovipositor (Fig. 5e) with tergite 10 pale brownish yellow. Cercus pale brownish yellow with dorsal area darker, distal part curved dorsally, tip acute. Sternite 8 yellow. Hypogynial valve pale brownish yellow with middle area yellow, tip reaching approximately 1/3 of cercus.

##### Distribution.

China (Guangxi, Zhejiang); South Korea; Japan.

##### Remarks.

Helius (H.) nipponensis is distributed in China, South Korea and Japan (Oosterbroek 2023). In China, this species was previously known in Zhejiang (Podenas et al. 2015) and is now recorded in Guangxi for the first time. For descriptions and illustrations of this species, also see Alexander (1913, 1920, 1929b) and Podenas et al. (2015).

#### ﻿ Helius (Helius) stenorhynchusstenorhynchus

Taxon classificationAnimaliaDipteraLimoniidae

Alexander, 1954

60D8C8EB-2FF8-5FBD-B70D-A551D517E0E6

Figs 7 , 8

Helius (Helius) stenorhynchus Alexander, 1954: 161. Type locality: Myanmar, Kambaiti.

##### Specimens examined.

China • 5 ♂, 2 ♀; Guangxi Zhuang Autonomous Region, Tianlin County, Cenwanglaoshan National Nature Reserve, Langping protection station; 1550 m a.s.l.; 23 May 2013; Guoquan Wang leg.; CAU.

##### Diagnosis.

Antenna with basal flagellomeres oval and crowded. Rostrum unusually long and slender, about equal in length to combined head (excluding rostrum) and thorax. Prescutum and presutural scutum brownish red with an indistinct darker median line, anterior region more or less infuscated. Femora of legs yellow with narrowly darker tips. Wing with oval brown stigma and inconspicuous dark seams; Sc atrophied, sc-r at its tip and ending slightly before fork of Rs; m-cu beyond fork of M. Outer gonostylus nearly straight; inner spine shorter and stouter. Interbase nearly globular with a curved, apically blackened spine-shaped outgrowth. Distal half of aedeagus curly dorsally.

##### Description.

**Figure 7.**
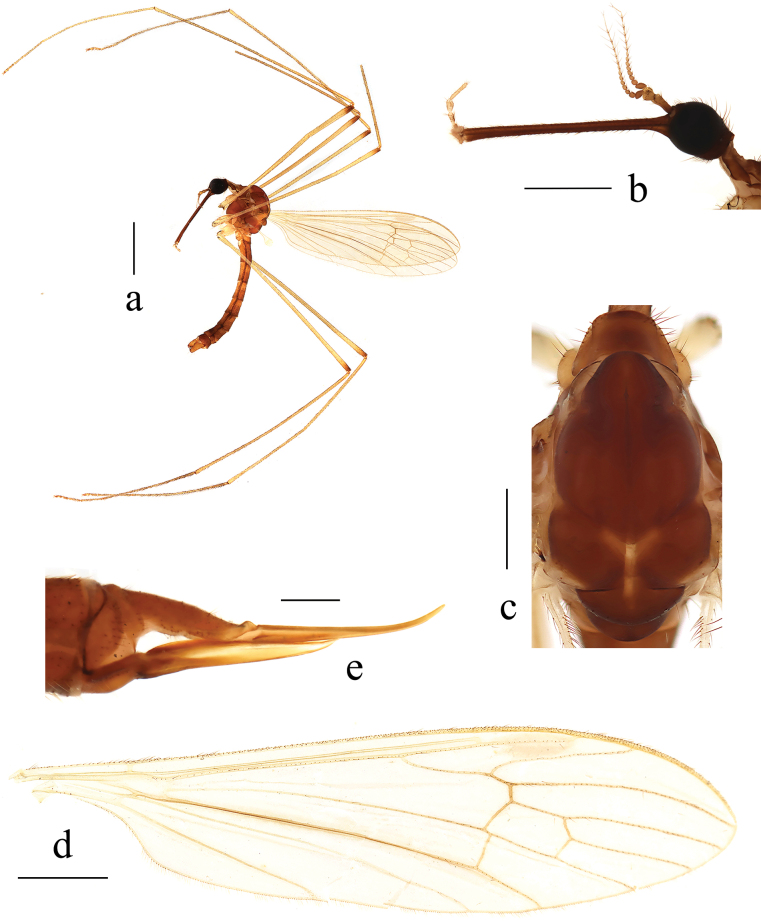
Helius (Helius) stenorhynchus stenorhynchus Alexander, 1954 **a** habitus of male, lateral view **b** male head, lateral view **c** male thorax, dorsal view **d** male wing **e** female ovipositor, lateral view. Scale bars: 2.0 mm (**a**); 1.0 mm (**b**); 0.5 mm (**c**); 1.0 mm (**d**); 0.4 mm (**e**).

**Male** (Fig. 7a). Body length 8.0–8.2 mm (excluding rostrum), wing length 7.8–8.0 mm, rostrum length 2.3–2.4 mm, antenna length 1.4–1.5 mm.

***Head*** (Fig. 7b). Brownish black. Setae on head dark brown. Antenna brown, scape and pedicel brownish black, flagellomeres brown with basal segments darker. Scape long cylindrical, 3 times as long as wide; pedicel nearly oval, widened distally; basal flagellomeres oval and crowded, with short brown verticils, outer flagellomeres tapering apically and elongated, with long brown verticils that exceed length of corresponding flagellomere. Rostrum unusually long and slender, about equal in length to combined head (excluding rostrum) and thorax, brownish black with dark brown setae. Palpus pale brownish yellow with pale brown setae.

***Thorax*** (Fig. 7c). Pronotum brownish red with middle darker. Prescutum and presutural scutum brownish red with an indistinct darker median line, anterior region more or less infuscated. Postsutural scutum brownish red, each lobe with a small white spot. Scutellum and mediotergite dark brown. Pleuron (Fig. 7a) dark brown. Setae on thorax dark brown. Fore and mid coxae pale yellow, hind coxa paler; trochanters pale yellow, tips narrowly black; femora yellow, tips narrowly darker; tibiae and tarsi dark yellow to pale brownish yellow. Setae on legs brown. Wing (Fig. 7d) tinged with grayish yellow. Stigma oval and brown; inconspicuous dark seams on cord, outer end of cell dm and CuA, darker on anterior cord. Veins pale brown to brown. Venation: Sc atrophied; sc-r at tip of Sc, ending slightly before fork of Rs; distance between tips of R_4_ and R_5_ 3–4 times as long as distance between tips of R_1_ and R_4_; cell dm about twice as long as wide; m-cu about 1/4 its length beyond fork of M. Halter yellow.

***Abdomen*** (Fig. 7a). Brown with first abdominal segment short and paler, segments 6–9 slightly darker; caudal border of each segment narrowly brownish black. Setae on abdomen dark brown.

**Figure 8.**
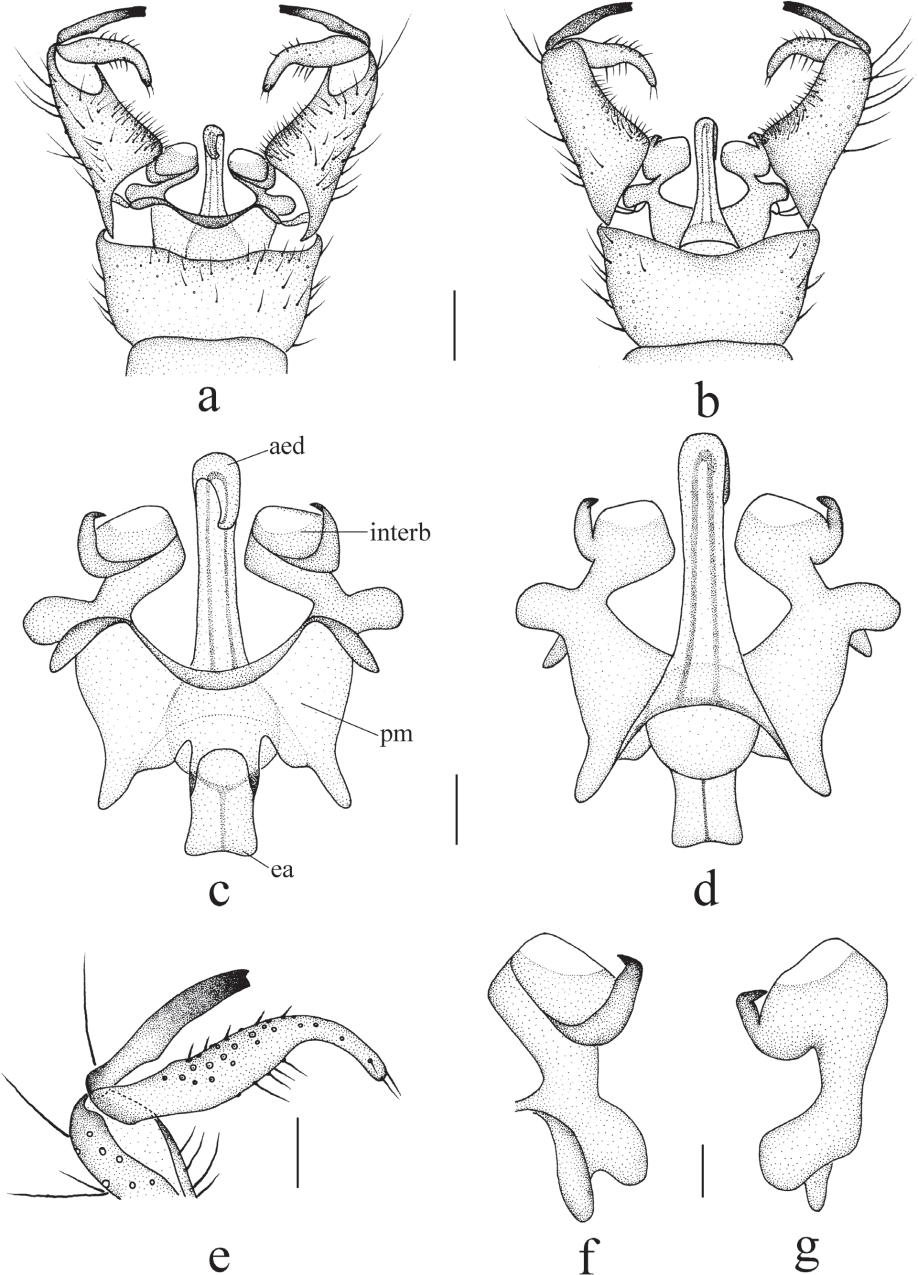
Helius (Helius) stenorhynchus stenorhynchus Alexander, 1954 **a** male hypopygium, dorsal view **b** male hypopygium, ventral view **c** aedeagal complex, dorsal view **d** aedeagal complex, ventral view **e** gonostyli, dorsal view **f** interbase, dorsal view **g** interbase, lateral view. Scale bars: 0.2 mm (**a, b**); 0.1 mm (**c, d, e**); 0.05 mm (**f, g**).

***Hypopygium*** (Fig. 8). Pale brown. Posterior margin of tergite 9 with a very shallow emargination (Fig. 8a). Gonocoxite nearly cylindrical, outer side with long brown setae (Fig. 8a, b). Outer gonostylus nearly straight, distal half blackened; tip weakly bispinous, inner spine shorter and stouter (Fig. 8a, b, e). Inner gonostylus arched, broad at base and gradually narrow, middle of outer side with short and stout pale brown setae, middle of inner side with long pale brown setae (Fig. 8a, b, e). Aedeagal complex with semen pump spherical (Fig. 8c, d); ejaculatory apodeme distinctly flattened, nearly rectangular (Fig. 8c, d); aedeagus with distal half curly dorsally (Fig. 8a–d). Parameres fused forming flattened transverse plate with short basal and apical parts, tip of apical part bent dorsally. Interbase nearly globular with a curved, apically blackened spine-shaped outgrowth (Fig. 8c, d, f, g).

**Female.** Body length 7.8–8.0 mm (excluding rostrum), wing length 7.6–8.0 mm, rostrum length 2.2–2.3 mm. Generally similar to male by body coloration. Ovipositor (Fig. 7e) with tergite 10 brown. Cercus brownish yellow to brown, long and slender, distal part curved dorsally, tip acute. Sternite 8 brown. Hypogynial valve yellow with base brown, tip slightly before middle of cercus.

##### Distribution.

China (Guangxi); Myanmar.

##### Remarks.

Helius (H.) stenorhynchus stenorhynchus was previously known only from Myanmar (Oosterbroek 2023) and is now recorded in China for the first time. For descriptions and illustrations of this subspecies, also see Alexander (1954).

## ﻿Conclusions

Here, the crane fly genus *Helius* is recorded in Guangxi for the first time with three species, of which H. (H.) damingshanus sp. nov. is described and illustrated as new to science, H. (H.) stenorhynchus stenorhynchus Alexander, 1954, previously known only from Myanmar, is recorded in China for the first time, and H. (H.) nipponensis (Alexander, 1913), previously known from Zhejiang, China, as well as South Korea and Japan, is also added to the fauna of Guangxi. The known species and subspecies are also redescribed and illustrated. An identification key to H. (Helius) crane flies in China is presented for the first time.

## Supplementary Material

XML Treatment for Helius

XML Treatment for Helius (Helius) damingshanus

XML Treatment for Helius (Helius) nipponensis

XML Treatment for Helius (Helius) stenorhynchusstenorhynchus

## ﻿Additional information

### Author ORCIDs

Yuanyuan Xu https://orcid.org/0009-0005-2118-0391

Shenglin Zhang https://orcid.org/0009-0009-5924-6113

Guoquan Wang https://orcid.org/0000-0003-1693-1654

Ding Yang https://orcid.org/0000-0002-7685-3478

Xiao Zhang https://orcid.org/0000-0002-8054-0968

### Data availability

All of the data that support the findings of this study are available in the main text.
